# Inhibition of the NLRP3 inflammasome provides neuroprotection in rats following amygdala kindling-induced status epilepticus

**DOI:** 10.1186/s12974-014-0212-5

**Published:** 2014-12-17

**Authors:** Xiang-Fei Meng, Lan Tan, Meng-Shan Tan, Teng Jiang, Chen-Chen Tan, Meng-Meng Li, Hui-Fu Wang, Jin-Tai Yu

**Affiliations:** Department of Neurology, Qingdao Municipal Hospital, School of Medicine, Qingdao University, Qingdao, PR China; Department of Neurology, Qingdao Municipal Hospital, Nanjing Medical University, Nanjing, PR China; Department of Neurology, Qingdao Municipal Hospital, College of Medicine and Pharmaceutics, Ocean University of China, Qingdao, PR China; Department of Neurology, Nanjing First Hospital, Nanjing Medical University, Nanjing, PR China; Department of Neurology, Memory and Aging Center, University of California, San Francisco, CA USA

**Keywords:** NLRP3, Inflammasome, Status epilepticus, Cytokine, IL-1β, IL-18, Caspase-1, Neuroinflammation, Spontaneous recurrent seizures, Hippocampal neuronal loss

## Abstract

**Background:**

NLRP3 inflammasome is proposed to regulate inflammation in several neurological diseases, but its role in epilepsy remains largely unknown. This study aimed to investigate the role of the NLRP3 inflammasome in neuroinflammation, spontaneous recurrent seizures (SRS) and hippocampal neuronal loss in rat brain following amygdala kindling-induced status epilepticus (SE).

**Methods:**

We detected the protein levels of IL-1β and NLRP3 inflammasome components by Western blot in the hippocampus of shams and SE rats at different time points following SE. To further examine whether the activation of the NLRP3 inflammasome contributes to SE-associated neuronal damage, we employed a nonviral strategy to knock down NLRP3 and caspase-1 expression in brain before undergoing SE. Proinflammatory cytokine levels and hippocampal neuronal loss were evaluated at 12 hours and at 6 weeks following SE respectively in these NLRP3 and caspase-1 deficient rats. Meanwhile, SRS occurrence was evaluated through a 4-week video recording started 2 weeks after SE in these NLRP3 and caspase-1 deficient rats.

**Results:**

IL-1β levels and NLRP3 inflammasome components levels dramatically increased at 3 hours after SE, and reached a maximum at 12 hours after SE compared with the control group. Knock down of NLRP3 or caspase-1 decreased the levels of IL-1β and IL-18 at 12 hours after SE, which was accompanied by a significant suppression in the development and severity of SRS during the chronic epileptic phase. Meanwhile, knock down of NLRP3 or caspase-1 led to a remarkable reduction of hippocampal neuronal loss in the CA1 and CA3 area of the hippocampus at 6 weeks after SE.

**Conclusions:**

Our study provides the first evidence that the NLRP3 inflammasome was significantly up-regulated following SE. More importantly, we show that inhibition of the NLRP3 inflammasome provides neuroprotection in rats following SE. These findings suggest that NLRP3 may represent a potential target for the treatment of epileptogenesis

**Electronic supplementary material:**

The online version of this article (doi:10.1186/s12974-014-0212-5) contains supplementary material, which is available to authorized users.

## Background

Status epilepticus (SE), which is one of the most serious manifestations of epilepsy, can be defined as 'a condition characterized by an epileptic seizure that is so frequent or so prolonged as to create a fixed and lasting condition' [1]. Brain inflammation promotes increased neuronal excitability, decreases seizure threshold and is likely to be involved in the molecular, structural and synaptic changes characterizing epileptogenesis [2]. Among all the enhanced proinflammatory cytokines following SE, IL-1β is regarded as a pivotal therapeutic target in SE, as observations have demonstrated IL-1β as being an important inflammation-related epileptogenic factor [3–5]. Many anti-seizure drugs are known to decrease IL-1β levels [6,7]. Moreover, the inhibition or deletion of caspase-1, the enzyme which cleaves pro-IL-1β producing the mature and biologically active form of IL-1β, attained significant seizure reduction [8,9]. However, the mechanisms by which the production of IL-1β is regulated have not been established.

The nucleotide binding and oligomerization domain-like receptor family pyrin domain-containing 3 (NLRP3) inflammasome is a multiprotein complex that mediates the activation of caspase-1, which in turn cleaves pro-IL-1β to form the mature IL-1β and is found to be a pivotal mediator of IL-1β function [10]. The NLRP3 inflammasome [11], composed of NLR family, pyrin domain containing 3 (NLRP3), apoptosis-associated speck-like protein containing a caspase recruitment domain (ASC) and caspase-1, mediates IL-1β transcription and functions via coupling with the NF-κB inflammatory pathway [12]. The NLRP3 inflammasome has been demonstrated as being associated with the innate immunity and inflammatory regulation of the central nervous system (CNS) [13–15]. It should be noted that the high concentrations of extracellular ATP and K^+^ ions, the generation of reactive oxygen species (ROS), the increased intracellular Ca^2+^ concentration, acidosis, hypoxia and cell swelling can activate NLRP3 [16–18]. Actually, all of the above factors underlie the generation of SE [19–22]. We hypothesized that this inflammasome may have potential to induce IL-1β-related neuroinflammation in SE. Therefore, it is intriguing to investigate the role of the NLRP3 inflammasome in SE-associated pathology and functional outcomes.

To test this hypothesis, we first investigated the expression profiles of IL-1β and NLRP3 inflammasome components, including NLRP3 and caspase-1 after SE. Next, we applied small interfering RNAs (siRNAs) to knock down NLRP3 and caspase-1 *in vivo*, and measured the alteration in proinflammatory cytokine and NLRP3 inflammasome components as well as the effects on functional outcomes.

## Methods

### Animals and experiments groups

To avoid the interference of estrogen on microglial activation, neuroinflammation and cognitive function [23], only male rats were used in this study. Adult male Sprague-Dawley (SD) rats weighing 260 to 300 g were obtained from the Experimental Animal Center of Qingdao University. The rats were specific-pathogen free rats, regularly checked to ensure the status, and housed in a pathogen-free room with a 12-hour light/dark cycle and given free access to food and water. All experiments were performed in strict accordance with the National Institute of Health Guide for the Care and Use of Laboratory Animals. Animal care and sacrifice were conducted according to methods approved by the Qingdao University Animal Experimentation Committee, and all efforts were made to minimize the number of animals used and their suffering. Experimental group allocation and experimental design are shown in Additional file 1: Figure S1.

### siRNA administration in rat brain

The NLRP3 siRNA was designed and synthesized by Invitrogen (Carlsbad, CA, USA). Caspase-1 siRNA and control siRNA were purchased from Santa Cruz Biotechnology (Santa Cruz, CA, USA). Entranster™-in vivo transfection reagent was purchased from Engreen Biosystem Co, Ltd (Beijing, China). We prepared the Entranster™-in vivo-siRNA mixture according to the manufacturer’s instructions. Briefly, 50 μg siRNA were resuspended in 50 μL RNase-free water to make a siRNA solution. Then, 50 μL of siRNA solution were mixed with 50 μL of Entranster™-in vivo transfection reagent and 100 μL artificial cerebrospinal fluid (aCSF, composition in mmol/L: NaCl 130, KCl 2.99, CaCl_2_ 0.98, MgCl_2_•6H_2_O 0.80, NaHCO_3_ 25, Na_2_HPO_4_•12H_2_O 0.039, NaH_2_PO_4_•2H_2_O 0.46) to obtain a 200 μL Entranster™-in vivo transfection mixture. Then, the 200 μL Entranster™-in vivo transfection mixture (containing NLRP3 or caspase-1 siRNA, control siRNA, no siRNA (aCSF only)) was filled into an osmotic pump (Model 2006; ALZET, Cupertino, CA, USA). Meanwhile, rats were anesthetized with 10% chloral hydrate (0.3 mL/100 g, intraperitoneal) and were placed in a stereotaxic apparatus (Stoelting, Wood Dale, IL, USA). A brain-infusion cannula (Brain Infusion Kit 2; ALZET, Cupertino, CA, USA) coupled via vinyl tubing to the osmotic pump was implanted into the dorsal third ventricle (AP: −1.8 mm; L: −0 mm; V: −5 mm). The osmotic pump was placed subcutaneously between the rat scapulae, and the siRNAs were continuously infused into the brain over a 6-week duration at a flow rate of 0.15 μL/hour. This dose of NLRP3 siRNA or caspase-1 siRNA infusion was well tolerated, and no signs of neurotoxicity including hind-limb paralysis, vocalization, food intake, or neuroanatomical damage were observed in preliminary study. Incidentally, the behavioral and biochemical data between control siRNA-treated and non-siRNA-treated rats do not differ. All of the operations were carried out with aseptic techniques.

### Electrode implantation and SE induction

SE was triggered following the methods of our previous work [24]. Briefly, rats were fixed in a stereotactic apparatus (Stoelting, Wood Dale, IL, USA) under deep anesthesia (10% chloral hydrate, 0.3 mL/100 g, intraperitoneal). The electrodes were permanently implanted into the right basolateral amygdala (AP: −3.0 mm; L: −4.8 mm; V: −8.8 mm) and were connected to a miniature receptacle, embedding in the skull with screws and dental acrylic cement. After electrode implantation, the animals were allowed to recover from surgery for 2 weeks. SE was induced by continuous delivery of 100-ms trains, consisting of 60 Hz 400 μA (peak-to-peak) bipolar 1-ms square-wave pulses, delivered at 60 Hz every 0.5 seconds using a ML1101 electronic stimulator (Nihon Kohden) via the implanted electrode for up to 20 minutes. Electroencephalograms (EEGs) of the right amygdala were recorded with a digital amplifier (AD Instrument, Bio Amp (Shanghai, China), USA). After 20 minutes of continuous stimulation the stimulation was interrupted and the behavioral and electrographic activity of the animals was observed for 60 seconds. If the behavior of the animals indicated the presence of epileptic activity (head nodding or limb clonus), observation was continued for another 5 minutes. If an animal did not meet the criteria of clonic SE (continuous EEG epileptiform spiking and recurrent clonic seizures), stimulation was resumed and the behavior of the animal was checked again after 5 minutes. Once the criteria of SE were achieved, no further stimulation was given. Stimulation period never exceeded 40 minutes. All of the operations were carried out with aseptic techniques. Sham rats were handled in the same manner but without receiving any electrical stimulation. Moreover, our preliminary experiments also show that this amygdala stimulation model is effective.

### Monitoring of SRS

SRS occurrence was evaluated through a 4-week video monitoring started 2 weeks after SE. All recordings for SRS were done during the light period. Epileptic rats were video-recorded for at least 12 hours daily. The frequency and duration of stage 4/5 seizures were recorded, and the severity of SRS was scored according to Racine’s scale [25]. The recordings were analyzed by observers who were blind to the results of group allocation.

### Brain tissue preparation

Rats were sacrificed under deep anesthesia and were handled as follows: For Western blot analysis, quantitative real-time PCR, and ELISA, rats were perfused transcardially with 0.9% saline (pH 7.4) only. The brains were removed rapidly and stored in liquid nitrogen until use. For cresyl violet staining and terminal deoxynucleotidyl transferase-mediated dUTP end-labeling assay (TUNEL) analysis, rats were perfused transcardially with 0.9% saline (pH 7.4), followed by a fixative solution containing 4% paraformaldehyde in 0.9% saline (pH 7.4). The brains were removed and fixed in the same fixative at 4°C until use. For double immunofluorescence staining, rat brain was removed without perfusion, embedded in tissue freezing medium, and immediately frozen at −4°C. Frozen tissue was stored at −80°C until sectioning.

### Western blotting

Tissues samples were digested with radio immunoprecipitation assay (RIPA) lysis buffer (50 mmol/L Tris-HCl, 150 mmol/L NaCl, 1% Nonidet-40, 0.5% sodium deoxycholate, 1 mmol/L EDTA, 1 mmol/L PMSF) with protease inhibitors (pepstatin 1 μg/mL, aprotinin 1 μg/mL, leupeptin 1 μg/mL) for 30 minutes and centrifuged at 12,000 × g for 15 minutes at 4°C. The protein concentration was determined using the Bradford assay kit (Bio-Rad Laboratories, Hercules, CA, USA). The equal amount of protein from different samples was separated using 8 to12% sodium dodecyl sulfate (SDS) polyacrylamide gels and transferred to polyvinylidene fluoride (PVDF) membranes. The membranes were blocked with 10% non-fat milk in Tween-TBS (TBST) and incubated at 4°C overnight, with the primary antibodies against NLRP3 (1:200; Santa Cruz Biotechnology, Santa Cruz, CA, USA), cleaved IL-1β (1:200; Santa Cruz Biotechnology, Santa Cruz, CA, USA), cleaved caspase-1 (1:200; Biorbyt, San Francisco, CA, USA), and β-actin (1:1,000; Santa Cruz Biotechnology, Santa Cruz, CA, USA). After rinsing, the membranes were appropriately incubated with horseradish peroxidase (HRP)-conjugated suitable secondary antibodies (1:5,000; Zhongshan Inc., Beijing, China) for 2 hours at room temperature. Cross-reactivity was visualized using electrochemiluminescence (ECL) Western blotting detection reagents and analyzed by scanning densitometry using a UVP BioDoc-It Imaging System (UVP, Upland, CA, USA).

### Quantitative Real-Time PCR

Total RNA was extracted using TRIzol reagent (Invitrogen, Carlsbad, CA, USA), using the protocol supplied by the manufacturer. The cDNA was synthesized using Reverse Transcription System (Bio-Rad, Hercules, CA, USA). The reaction was performed at 42 °C for 50 minutes, 95°C for 5 minutes, and 5°C for 5 minutes, then the cDNA was stored at −20°C. Amplification was carried out using the Stratagene Mx3000P real-time PCR system (Stratagene, La Jolla, CA, USA) and real-time SYBR Green PCR technology (Takara Bio, Inc., Shiga, Japan). Reverse transcription was performed in a final volume of 20 μL containing 2 μL cDNA, 10 μL SYBR Green, 0.4 μL ROX Reference Dye, 0.4 μL forward and reverse primer (1 mol/L), and 6.8 μL nuclease-free water. The optimal conditions were 40 cycles of 95°C for 30 seconds, 60°C for 32 seconds, and 72°C for 30 seconds. All reactions were run in duplicates and the mean values are used. Total RNA concentrations from each sample were normalized by quantity of β-actin mRNA, and the target genes’ expression was evaluated by ratio of the number of target mRNA to β-actin mRNA. Relative expression of genes was obtained by the 2^-△△CT^ method. Primers were purchased from Invitrogen (Carlsbad, CA, USA) as follows (name: forward primer, reverse primer): nlrp3: 5’-ccagggctctgttcattg-3’, 5’-ccttggctttcacttcg-3’; caspase-1: 5’-aggagggaatatgtggg-3’, 5’-aaccttgggcttgtctt-3’; β-actin: 5’-agggaaatcgtgcgtgac3’, 5’-cgctcattgccgatagtg-3’.

### Cytokine measurement

The cytokine analysis was performed in duplicate using commercial ELISA assay kits according to the manufacturers’ instructions. IL-1β was measured in ELISAs from R&D Systems (Minneapolis, MN, USA). IL-18 was measured in ELISAs from Invitrogen (Carlsbad, CA, USA). The results are expressed in pg/mL.

### Nissl staining (cresyl violet staining) and TUNEL staining

The brains were embedded in paraffin and cut into 7-μm sections. Nissl staining was employed to detect surviving neurons. Briefly, the paraffin-embedded sections were dewaxed and rehydrated according to the standard protocols, and immersed in 1% cresyl violet at 50°C for 5 minutes. After being rinsed with water, the sections were dehydrated in increasing concentrations of ethanol, mounted on the slides, and examined with a light microscope. Only the neurons with a violet nucleus and intact morphology were counted as surviving neurons. The TUNEL staining, which detects DNA fragmentation resulting from apoptotic signaling cascades, was performed to label apoptotic neurons. It may also label cells that have suffered severe DNA damage. Therefore, the TUNEL assay is helpful in identifying seizure-induced neuronal loss in our experiment. We employed the TUNEL assay via a commercial kit according to the manufacturer's instructions (Roche Co., Mannheim, Germany). TUNEL-positive neurons with condensed nuclei were identified as dead neurons. Cell counting was performed on six randomly selected non-overlapping fields in the CA1 and CA3 regions of the hippocampus per slide. The densities of surviving neurons or TUNEL-positive neurons in the hippocampus of the scanned digital images were calculated using Image-Pro Express software (Media Cybernetics, Silver Spring, MD, USA). The total cell counts were averaged from six sections per animal. The survival index was defined as:

$$ Surviving\kern0.5em  index\kern0.5em \left(\%\right)=\kern0.5em 100\times \left( Number\kern0.5em  of\kern0.5em  survivng\kern0.5em  neurons/ Total\kern0.5em  numbers\kern0.5em  of\kern0.5em  neurons\right) $$

Furthermore, the TUNEL-positive neuron index was defined as:

$$ TUNEL- positive\kern0.5em  neurons\kern0.5em  index\kern0.5em \left(\%\right)=100\times \left( Count\kern0.5em  of\kern0.5em  TUNEL- positive\kern0.5em  neurons/ Total\kern0.5em  count\kern0.5em  of\kern0.5em  neurons\right) $$

### Double immunofluorescence staining

In brief, frozen tissue sections of hippocampus area (20 μm thick) from epileptic rats at 12 hours following amygdala stimulation and time-matched shams were used for double staining of NLRP3/ionized Ca^+^ binding adaptor molecule 1 (Iba1). The sections were obtained by cryosectioning at −20°C, mounted on a glass slide, and incubated at room temperature for 1 hour. Afterward, the sections were fixed in ice-cold acetone for 10 minutes and then dried on a heater for 10 minutes at 40°C. The sections were then blocked with 5% BSA and 0.1% Triton X-100 for 2 hours at room temperature. After a single wash with PBS, sections were incubated overnight at 4°C with a goat polyclonal antibody against Iba1 (1:100, Abcam, Cambridge, UK) as well as a rabbit polyclonal antibody against NLRP3 (1:50; Santa Cruz Biotechnology, Santa Cruz, CA, USA)). Sections were rinsed in PBS and washed 3 times, and then incubated respectively with tetramethylrhodamine (TRITC)-conjugated anti-goat IgG (1:200; Zhongshan Inc., Beijing, China) and fluorescein isothiocyanate (FITC)-conjugated anti-rabbit IgG (1:200; Santa Cruz Biotechnology) for 2 hours at room temperature in a dark and humidified container. After that, the sections were washed with PBS and sealed with a coverslip. The slides were analyzed with a fluorescence microscopy (Olympus, Tokyo, Japan). For nuclear staining we used 4',6-diamidino-2-phenylindole (DAPI). To ensure the specificity of the immunoblotting procedure, control experiments were performed in which the corresponding primary antibody was omitted. Under these conditions, no signal was observed.

### Statistical analysis

Statistical analysis was carried out by SPSS software 17.0 (IBM Inc., Chicago, IL, USA). After confirming normal distribution with skewness and kurtosis statistic test, independent sample *t*-test or one-way analysis of variance (ANOVA) followed by the Bonferroni *post hoc* test used to analyze differences among groups. All data were presented as mean ± standard deviation (SD). *P* < 0.05 was considered statistically significant.

## Results

### Cleaved IL**-**1β, NLRP3 and cleaved caspase**-**1 were up**-**regulated in SE rat

**Figure 1:**
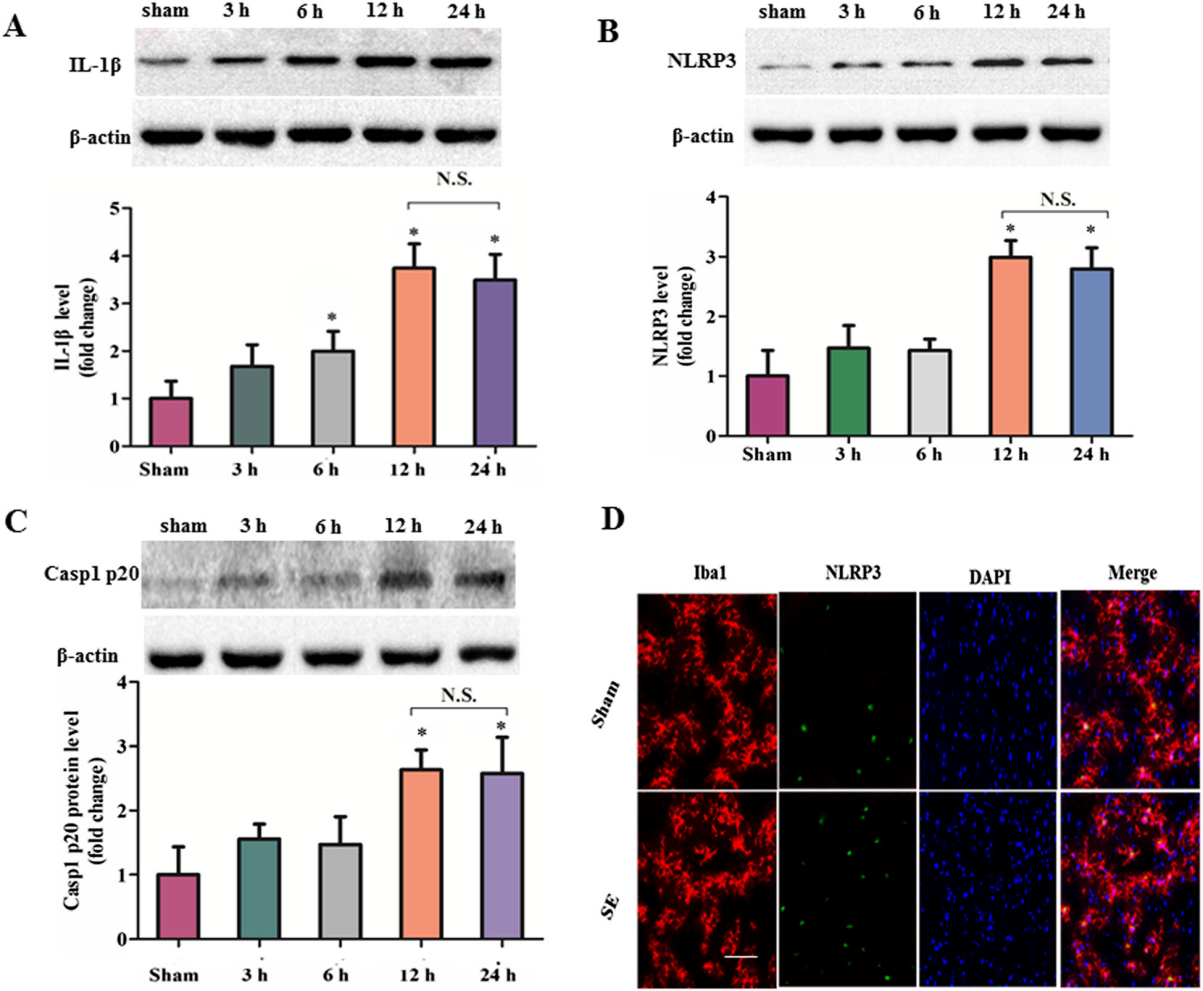
**Expression profiles of the nucleotide binding and oligomerization domain-like receptor family pyrin domain-containing 3 (NLRP3) inflammasome components following status epilepticus (SE). (A-C)** Western blot assay for the expression profiles of cleaved IL-1β (18 kDa) **(A)**, NLRP3 (106 kDa) **(B)** and cleaved caspase-1 (20 kDa) **(C)** in the hippocampus of sham rat at 12 hours following the sham operation of amygdala stimulation (without any electrical stimulation) and SE rat at 3, 6, 12, and 24 hours following amygdala stimulation. Data are expressed as a fold change relative to sham group. n = 6 rat per group and per time point. Error bars represent mean ± standard deviation. *P < 0.05 versus sham control group. NS: not significant versus 12 hours following SE. **(D)** Representative photographs of immunofluorescence staining for NLRP3 (green) expression in microglia (Iba-1, red) in the hippocampal area 12 hours following SE. Scale bars: 100 μm.

We first investigated whether the expression of IL-1β was altered in rat brain after SE which was induced by amygdala stimulation. Total proteins were extracted from the hippocampal regions of SE rats at different time points (including 3, 6, 12, 24 hours following SE) and of shams, and subjected to Western blot analysis. Because there was no significant difference in all detected variables among different sham groups according to our preliminary analysis, the samples of sham group were collected at 12 hours after the sham operation of amygdala stimulation (without any electrical stimulation). We found that the cleaved IL-1β levels of SE rats were significantly elevated compared to control samples during all time points studied (Figure 1A). In the SE group, cleaved IL-1β levels dramatically increased at 3 hours and reached a maximum at 12 hours (about 4 × those of the sham group ) after SE (*P* < 0.05; n = 6). Following this maximum, no statistically significant difference was observed in expression level of cleaved IL-1β among rat brains at 24 hours compared with that at 12 hours (*P* = 1.000; n = 6). Next, we analyzed the expression of hippocampal NLRP3 inflammasome components in SE rats. Importantly, the levels of hippocampal NLRP3 protein, and caspase-1 p20, the products of NLRP3 inflammasome activation, were significantly increased at 3 hours and reached a maximum at 12 hours after SE (both *P* < 0.05; n = 6, Figure 1B, C). Accordingly, no significant differences were observed in NLRP3 levels (*P* = 1.000; n = 6), and cleaved caspase-1 levels (*P* = 1.000; n = 6) among rat brains at 24 hours compared with that at 12 hours after SE. Using double immunofluorescence staining to colocalize NLRP3 with microglia marker Iba1, our result further demonstrated the increased expression of NLRP3 in the Iba1-positive microglia of hippocampus at 12 hours following SE (Figure 1D).

### **Downregulation of NLRP3 by siRNA led to significant reduction in proinflammatory cytokines and cleaved caspase**-**1 expression levels in SE rat**

To directly study the potential impact of increased NLRP3 expression levels on the maturation and secretion of IL-1β in SE rats, we knocked down brain NLRP3 expression in animals before undergoing SE by using *in-vivo* nonviral RNA interference methodology [26]. To evaluate the silencing efficiency of siRNA infusion, the gene expression and protein level of NLRP3 protein were detected by quantitative real-time PCR and Western blotting, respectively. This approach of using NLRP3 siRNA infusion could produce a significant down-regulation of NLRP3 mRNA (by 60%) and protein levels (by 51%) in the brain compared with control siRNA under epileptic conditions (all *P* < 0.05; Additional file 2: Figure S2 A, B). Incidentally, the NLRP3 levels and gene expression between control-siRNA treated and non-siRNA-treated tissues under epileptic conditions or under control conditions do not differ (all *P* > 0.05; n = 6, Additional file 2: Figure S2 A, B, and E), excluding potential indirect siRNA-effects on gene expression or the protein level of NLRP3.

**Figure 2:**
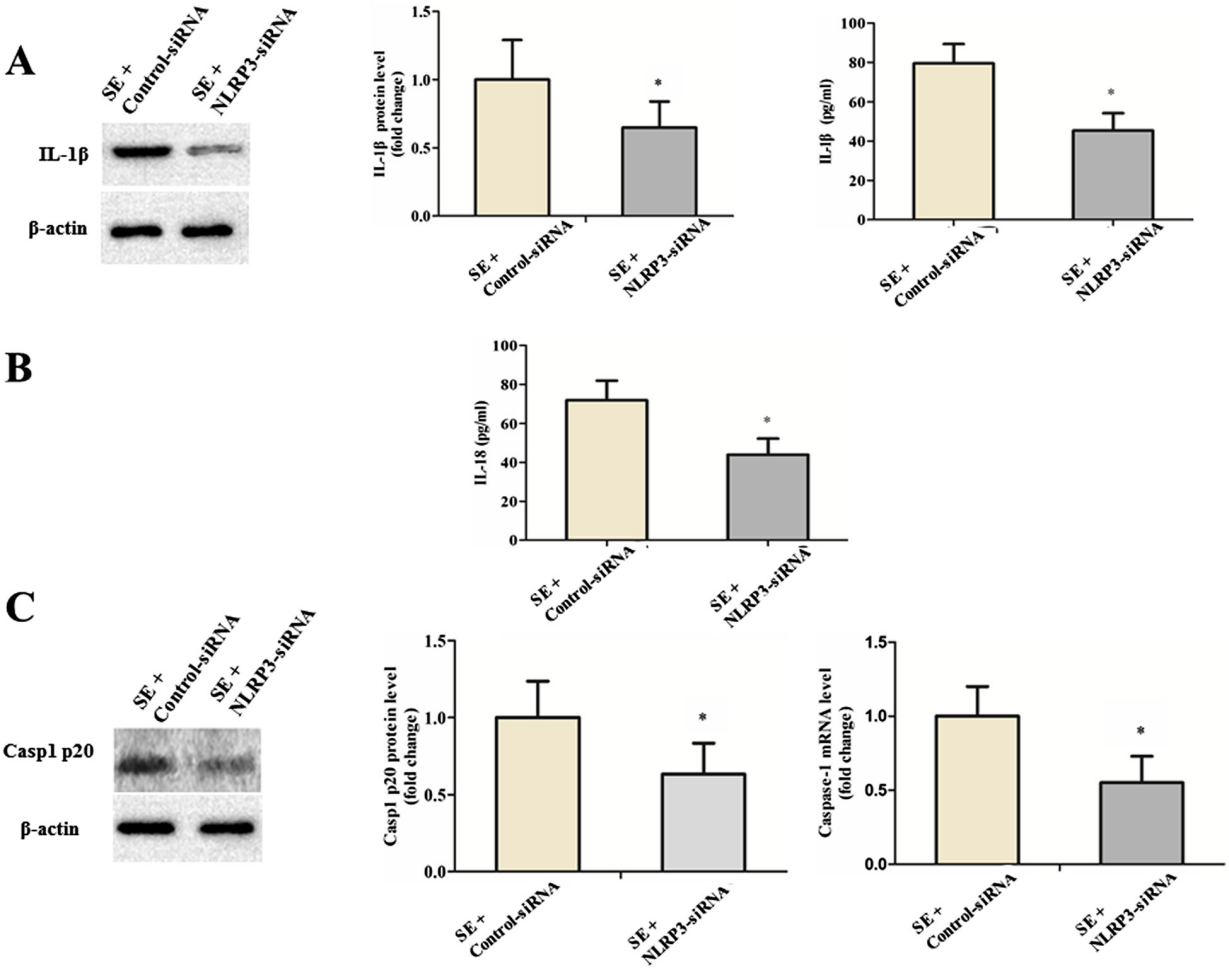
**Downregulation of nucleotide binding and oligomerization domain-like receptor family pyrin domain-containing 3 (NLRP3) by siRNA led to significant reduction of proinflammatory cytokines and cleaved caspase-1. (A)** The expression level of cleaved IL-1β (18 kDa) was detected by Western blot analysis and ELISA. β-actin was used as loading control. **(B)** The expression level of cleaved IL-18 was detected by ELISA. **(C)** The protein expression level of active caspase-1 (20 kDa) was analyzed using the Western blot assay. The gene expression level of caspase-1 was detected by quantitative real-time PCR. Data are expressed as a fold change relative to SE rat infused with control siRNA. All data are shown as mean ± standard deviation (n = 6 per group). *P < 0.05 versus control siRNA treatment.

Next, we determined the effects of NLRP3 knock down on SE-induced neuroinflammation. The proinflammatory cytokines expression levels were then assessed by Western blot analysis and ELISA assay. Compared to the control-siRNA group, the elevated expression level of cleaved IL-1β in the brain tissues of SE rats could be inhibited by NLRP3 siRNA treatment (*P* < 0.05; n = 6; Figure 2A). In addition, SE rats infused with NLRP3 siRNA showed a dramatic reduction in brain IL-18 protein levels (*P* < 0.05; n = 6; Figure 2B). We further examined the changes in caspase-1, which has been known to play a central role in the cleavage of IL-1β and IL-18. The gene expression and protein level of caspase-1 protein were detected by quantitative real-time PCR and Western blotting, respectively. As indicated in Figure 2C, the NLRP3 siRNA treatment could reduce the active caspase-1 protein levels and gene expression in SE rats (both *P* < 0.05; n = 6).

### Downregulation of NLRP3 by siRNA attenuated the development and severity of spontaneous recurrent seizures following SE

In above work, we demonstrated that NLRP3 mediated IL-1β over-expression and inflammatory signal activation in the SE rat hippocampus. Accumulating data suggest that inflammation may contribute to epileptogenesis in experimental models as well as in humans. However, whether anti-inflammatory treatments can prevent epileptogenesis remains controversial. Here, we examined the anti-epileptogenic effect of NLRP3 inhibition.

To determine the effect of NLRP3 siRNA on the development and severity of SRS in the chronic phase, SRS were observed from a 4-week video recording, which started 2 weeks after SE. The severity of SRS between siRNA (non or control)-treated SE rats and SE rats do not differ (all *P* > 0.05; n = 18, Additional file 3: Table S1), excluding an effect of siRNA transfection on status severity.

As revealed by Table 1(A), all rats (18/18) in the control siRNA-treated SE group had SRS during the monitoring period, whereas 66% (12/18) of the rats in the NLRP3 siRNA-treated SE group had SRS. The time to development of SRS was longer in the NLRP3 siRNA-treated SE group compared with the control siRNA-treated SE group (SE + control siRNA group: 14.8 ± 5.5 days; SE + NLRP3 siRNA group: 27.5 ± 9.3 days, Table 1A). The mean number of seizures (total number of seizures/number of recording days, calculated separately for each animal) in the control siRNA-treated SE group and NLRP3 siRNA-treated SE group was 7.58 ± 1.09 and 1.34 ± 0.44 seizures/day respectively. NLRP3 siRNA treatment also dramatically reduced the duration of observed seizures during the chronic epileptic phase (control siRNA, 26.62 ± 6.94 seconds; NLRP3 siRNA, 10.48 ± 2.76 seconds, Table 1A). The differences between the two groups were significant (*P* < 0.05, Table 1A).

**Table 1:** **The development and severity of animals with spontaneous recurrent seizures (SRS) during the 4-week video monitoring in experimental groups**

**(A) Results from rats after 6 weeks of treatment with NLRP3 siRNA or control siRNA**				
	**The number of rats developing SRS**	**The time to development of SRS (days)**	**The mean number of seizures (seizures/day)**	**The mean seizure duration (sec/seizure)**
Control siRNA + sham	0 (18)	0	0	0
NLRP3 siRNA+ sham	0 (18)	0	0	0
Control siRNA + SE	18 (18)	14.8 ± 5.5	7.58 ± 1.09	26.62 ± 6.94
NLRP3 siRNA+ SE	12 (18)^a^	27.5 ± 9.3^a^	1.34 ± 0.44^a^	10.48 ± 2.76^a^
**(B) Results from rats after 6 weeks of treatment with caspase-1 siRNA or control siRNA**				
	**The number of rats developing SRS**	**The time to development of SRS (days)**	**The mean number of seizures (seizures/day)**	**The mean seizure duration (sec/seizure)**
Control siRNA + sham	0 (18)	0	0	0
Caspase-1 siRNA + sham	0 (18)	0	0	0
Control siRNA+ SE	17 (18)	16.6 ± 6.1	9.02 ± 1.56	23.67 ± 5.90
Caspase-1 siRNA+ SE	11 (18)^a^	26.8 ± 9.1^a^	2.40 ± 0.78^a^	8.21 ± 2.66^a^

All data are shown as mean ± standard deviation (n = 18 per group). ^a^ *P* < 0.05 versus control siRNA treatment.

NLRP3, nucleotide binding and oligomerization domain-like receptor family pyrin domain-containing 3; SE, status epilepticus; sec, seconds; SRS, spontaneous recurrent seizures.

### Downregulation of NLRP3 by siRNA inhibited hippocampal neuronal loss in SE rat

**Figure 3:**
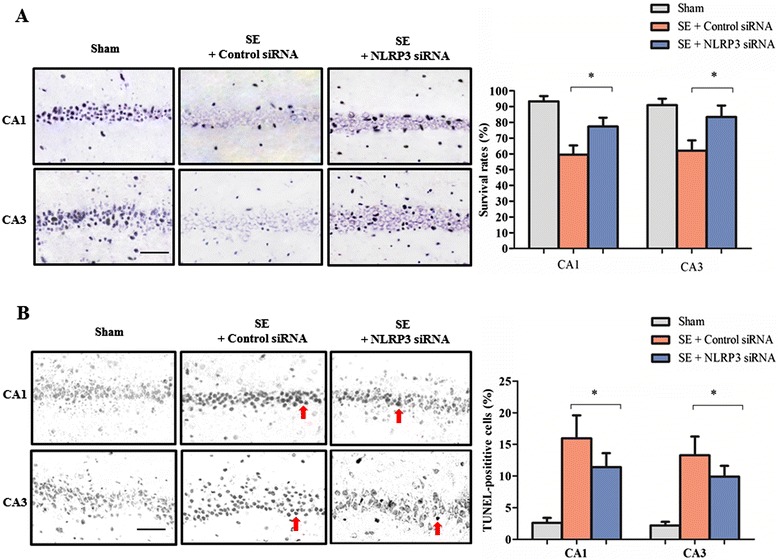
**Downregulation of nucleotide binding and oligomerization domain-like receptor family pyrin domain-containing 3 (NLRP3) by siRNA attenuated hippocampal neuronal loss in status epilepticus (SE rat). (A)** Representative photo of Nissl-staining in CA1 region and CA3 region of the rat hippocampus. Neurons with intact morphology were identified as surviving neurons. Scale bars: 50 μm. The neuronal survival rate was defined as follows: Neuronal surviving rate (%) = 100 × (Count of surviving neurons/Total count of neurons). **(B)** Neuronal death was detected using the TUNEL staining in the hippocampus of sham rats and SE rats. Photos were converted to black and white to obtain a better contrast ratio. Neurons with deep black nuclei were identified as TUNEL-positive neurons (indicated by red arrows). Scale bars: 50 μm. The percentage of TUNEL-positive neurons was defined as follows: 100 × (Count of TUNEL-positive neurons/Total count of neurons). Columns represent mean ± standard deviation (n = 6 per group). *P < 0.05 versus control siRNA treatment. TUNEL, terminal deoxynucleotidyl transferase-mediated dUTP end-labeling.

If left untreated, SE can cause irreversible brain damage. On this basis, we next investigated the effects of NLRP3 inhibition on neuronal loss in the CA1 and CA3 area of the hippocampus at 6 weeks after SE. Nissl staining was firstly used to detect surviving neurons in the hippocampus. As shown by Figure 3A, a significant increase in neuronal survival rate was noted in CA1 and CA3 regions in the hippocampus of SE rats treated with NLRP3 siRNA compared to the control-siRNA group (CA1: 77.34 ± 5.7% versus 59.57 ± 5.8%, *P* < 0.05; CA3: 83.43 ± 7.3% versus 62.1 ± 6.5%, *P* < 0.05). The TUNEL staining assay was then used. As indicated by Figure 3B, a dramatic reduction of the TUNEL-positive neurons index was observed in CA1 and CA3 regions of the hippocampus of SE rats treated with NLRP3 siRNA compared to the control-siRNA group (CA1: 11.41 ± 2.4% versus 15.95 ± 3.3%, *P* < 0.05; CA3: 9.9 ± 1.9% versus 13.29 ± 2.8%, *P* < 0.05).

### Downregulation of caspase**-**1 by siRNA alleviated neuroinflammation, spontaneous recurrent seizures and hippocampal neuronal loss following SE

**Figure 4:**
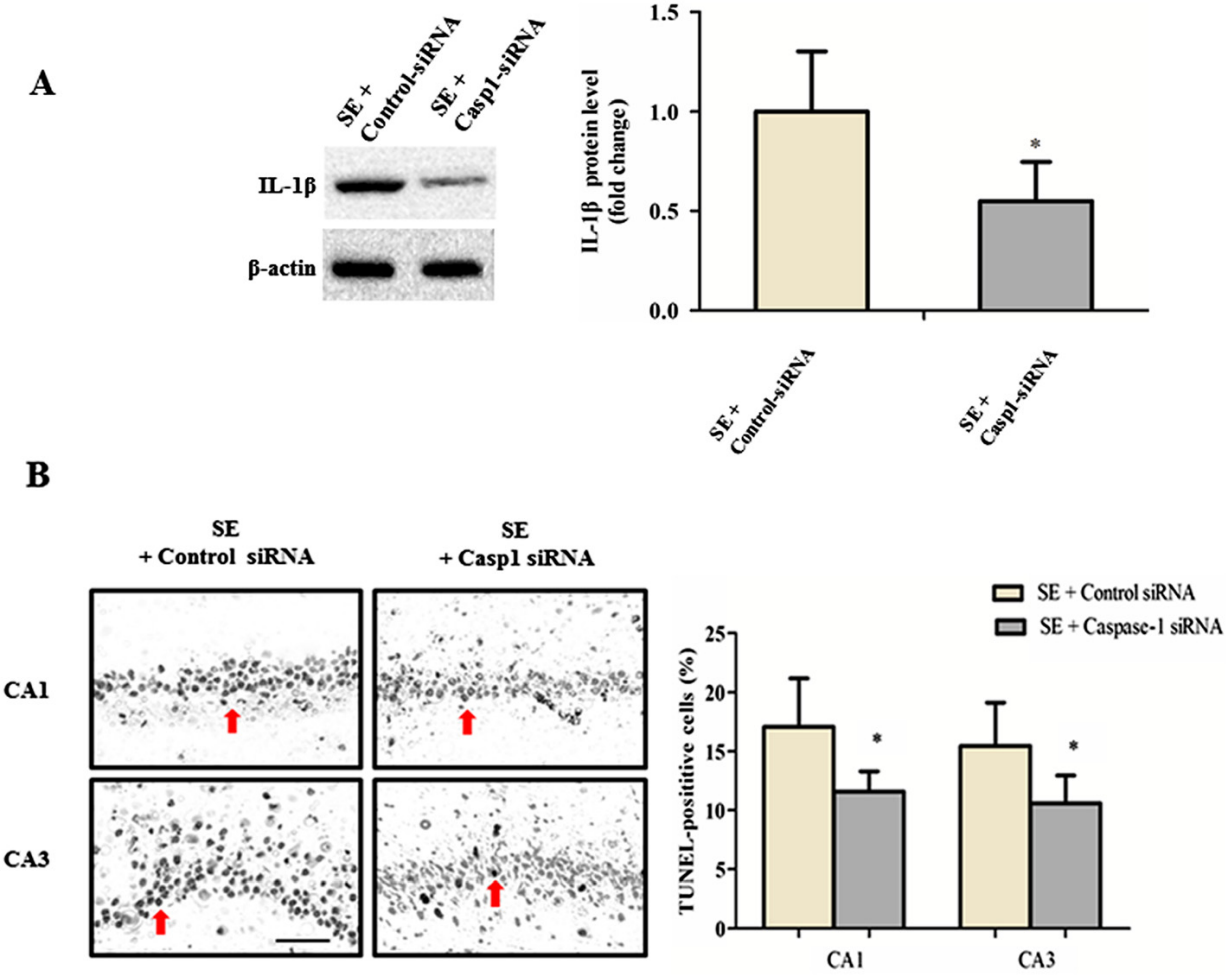
**Caspase-1 inhibition ameliorated neuroinflammation and hippocampal neuronal loss in status epilepticus (SE) rat. (A)** The expression level of cleaved IL-1β (18 kDa) was detected by Western blot. β-actin was used as loading control. Data are expressed as a fold change relative to SE rat infused with control siRNA. *P < 0.05 versus control siRNA treated group. **(B)** Neuronal death was detected using the TUNEL staining in the CA1 and CA3 region of the hippocampus. Photos were converted to black and white to obtain a better contrast ratio. Neurons with deep black nuclei were identified as TUNEL-positive neurons (indicated by red arrows). Scale bars: 50 μm. The percentage of TUNEL-positive neurons was defined as follows: 100× (Count of TUNEL-positive neurons/Total count of neurons). All data are shown as mean ± standard deviation (n = 6 per group). *P < 0.05 versus control siRNA-treated group. TUNEL, terminal deoxynucleotidyl transferase-mediated dUTP end-labeling.

Considering the central role of caspase-1 in the process of IL-1β maturation and secretion, we also successfully knocked down brain caspase-1 in SE rat by *in-vivo* nonviral RNA interference methodology for 6 weeks (Additional file 2: Figure S2 C, D, and F). We found a significant reduction of IL-1β levels after infused with caspase-1 siRNA (*P* < 0.05; n = 6, Figure 4A). Furthermore, caspase-1 silencing attenuated the development and severity of SRS in the chronic phase (*P* < 0.05; n = 18, Table 1B). A marked reduction of TUNEL-positive cell densities in the CA1 and CA3 region of the hippocampus in the SE rat treated with caspase-1 siRNA was also observed (*P* < 0.05; n = 6, Figure 4B).

## Discussion

Experimental evidence supports a role for inflammatory processes in the precipitation and recurrence of seizures and neuronal damage. The involvement of proinflammatory cytokine IL-1β in the development of seizure is strongly supported by pharmacological and genetic studies in animal models, showing that interference with IL-1β reduces the incidence of SE or attenuates recurrent seizures [8,27,28], whereas its amplification exacerbates seizures [27,29,30] as well as lowers the seizure threshold [15,31]. Accordingly, selective blockade [8,9], or gene deletion [9] of caspase-1, the enzyme which cleaves pro-IL-1β producing the mature and biologically active form of IL-1β, reduces seizures significantly. IL-1β could also affect neuronal excitability at different levels [32–34]. Both hyperexcitability and excitotoxicity are required and need to be sufficient for the contributory effect of IL-1β to the generation of SRS. Moreover, specific cytokines, including IL-1β, have been shown to contribute to neuronal death [35], perhaps in part via enhanced excitability [5]. Conversely, SE, in the absence of pre-existing or concomitant systemic or CNS inflammation, induce proinflammatory reactions in the brain (that is sterile inflammation), which in turn contribute to seizure recurrence and severity [36].

**Figure 5:**
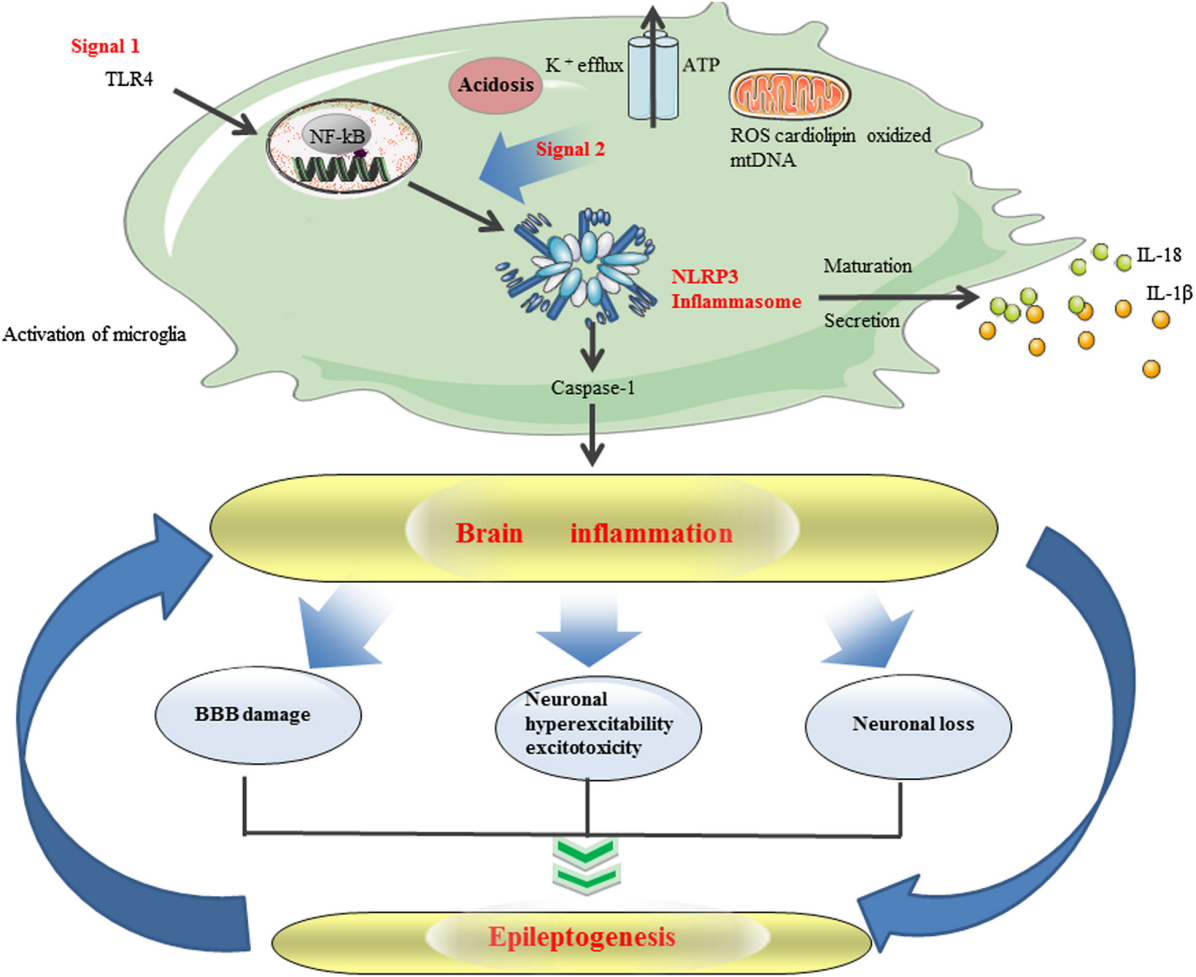
**A schematic linking the nucleotide binding and oligomerization domain-like receptor family pyrin domain-containing 3 (NLRP3) inflammasome activation to status epilepticus (SE) pathogenesis.** Activation of the NLRP3 inflammasome typically requires a bimodal signaling pathway. A Toll-like receptor (TLR)-dependent priming step activates the NF-κB-dependent transcription of NLRP3 and the pro-forms of the proinflammatory cytokines (which are IL-1β and IL-18). NLRP3-activating stimulation agents provide a second signal in the form of K^+^ efflux, cytosolic release of mitochondria-derived factors such as reactive oxygen species (ROS), cardiolipin, and oxidized mitochondrial DNA (mtDNA). Note that acidic extracellular pH represents a novel stimulation agent for triggering NLRP3 inflammasome activation. Oligomerization of NLRP3 is followed by recruitment of the adaptor molecule apoptosis-associated speck-like protein containing a caspase recruitment domain (ASC) and the pro-form of caspase-1, leading to the activation (cleavage) of caspase-1. Activated caspase-1 in turn catalyzes the cleavage of IL-1β and IL-18. This event may lead to changes in brain parenchyma such as leakage of the blood-brain barrier (BBB), neuronal hyperexcitability and excitotoxicity as well as neuronal damage which contribute to lowering the threshold for seizure induction and thus to trigger epileptogenesis. Activation of innate immune mechanisms during epileptogenesis can recruit inflammatory cells from the periphery which perpetuate inflammation, thus activating a vicious cycle that in turn fosters aberrant hyperexcitability. The onset of SE can in turn further promote inflammation via the production of proinflammatory cytokines.

Therefore, inflammation is both a cause and a consequence of SE. Experimental studies show that once SE develop, it can contribute to perpetuate inflammation in the brain, thus activating a vicious cycle that in turn fosters aberrant hyperexcitability (Figure 5). Thus, a prolonged condition of ongoing seizures with SE can be generated.

Our study was to examine for first time whether the NLRP3 inflammasome is a potential mechanism in neuroinflammation of SE rats. We compared expression of proinflammatory cytokine IL-1β, NLRP3 and caspase-1 in hippocampus from SE rats model evoked by amygdala stimulation to matched sham samples. The present study detected up-regulated IL-1β, NLRP3 and caspase-1 levels within SE samples than controls. All protein levels reach a maximum at 12 hours following SE. Meanwhile, the cellular localization of NLRP3 on microglia in SE rat brain was demonstrated by double immunofluorescence staining. Moreover, we knocked down brain NLRP3 expression by implanting mini-osmotic pumps for direct infusion of siRNA to investigate its role on neuroinflammation in the SE rat model. In our study, we found that this approach effectively down-regulated the levels of NLRP3 mRNA and protein in SE rat brain. Meanwhile, compared to non-siRNA-treated SE rat, the treatment with control siRNA did not alter NLRP3 mRNA and protein levels, thus excluding an effect of pump-mediated infusion on NLRP3 expression levels. For the first time, we revealed that NLRP3 siRNA treatment could significantly reduce proinflammatory cytokine levels and the active caspase-1 expression levels. Meanwhile, NLRP3 inhibition could also suppress SRS, and attenuate hippocampal neuronal loss.

As we know, caspase-1 is a critical pathway by which NLRP3 inflammasomes contribute to the downstream effects. Hence, we also knocked down brain caspase-1 by this *incpyvivo* nonviral RNA interference methodology in SE rats. Consistent biochemical and behavioral results were found between caspase-1 siRNA- and NLRP3 siRNA-treated SE rats, further supporting that NLRP3 exerts the effects of neuroinflammation in rats following SE.

We have determined that the NLRP3 inflammasome contributes to the SE-induced inflammatory response; however, the molecular basis of NLRP3 inflammasome activation in SE-induced brain injury has not been established. Evidence shows that assembly of the NLRP3 inflammasome depends on the activation of NLRP3. This process relies on exposure to whole pathogens, as well as a number of structurally diverse pathogen or danger-associated molecular patterns (PAMPs or DAMPs, respectively) and environmental irritants. It should be noted that, the high concentrations of extracellular ATP and K^+^ ions, and the generation of ROS are the most important factors for activating NLRP3 [37]. In fact, all of the factors above can be involved after SE. Additionally, it should be noted that IL-1β may trigger the classical cascade of events which includes the activation of the NF-κB-dependent pathways, thus resulting in the transcription of genes that may contribute to the acquired molecular changes (for example, modifications in ion channels) associated with the epileptogenic process [38]. Interestingly, NLRP3, an important component of the NLRP3 inflammasome complex, is activated by IKKβ/NF-κB [12]. The activation of the NLRP3 inflammasome, results in the secretion of bioactive IL-1β, and subsequently triggers the activation of NF-κB which in turn promotes the secretion of bioactive IL-1β [39]. Local acidosis has been demonstrated at inflammatory sites. Recent data suggest that acidosis is a regulator of inflammatory pathways [16,40]. The study by Edye *et al*. [40] suggested that acidosis promotes alternative DAMP-induced processing of IL-1β independent of caspase-1, and this result seemed to be inconsistent with the findings by Rajamäki and colleagues [16], as they found that acidic extracellular pH triggers NLRP3 inflammasome activation and IL-1 secretion in human macrophages (Figure 5).

## Conclusions

Our study firstly demonstrates that the expression of the NLRP3 inflammasome was up-regulated in the SE rat. The increase in NLRP3 levels can activate caspase-1 signaling that is responsible for neuroinflammation, neuronal loss and epileptogenesis (Figure 5). Using the pump-mediated *in vivo* infusion of nonviral siRNA to knock down NLRP3 and caspase-1 in the brain of SE rats, our study further indicated that inhibition of the NLRP3 inflammasome may play a neuroprotective role against SE-related neuroinflammation and neuronal damage.

## Additional files

Additional file 1: Figure S1. Scheme of the experimental design and the main experimental protocol. The rats were randomly divided into five groups: sham group, SE group, control siRNA + SE group, NLRP3 siRNA + SE group, and caspase-1 siRNA + SE group. siRNA, small interfering RNA; SE, status epilepticus; TUNEL assay, terminal deoxynucleotidyl transferase-mediated dUTP end-labeling assay; Elisa, enzyme-linked immunosorbent assay; qRT-PCR, quantitative real-time PCR. Additional file 2: Figure S2. Small interfering RNA (siRNA) targeting NLRP3 or caspase-1 effectively downregulated NLRP3 or caspase-1 in status epilepticus (SE) rat. (A) Messenger RNA levels of NLRP3 in brain of SE rats after 6-week infusion of artificial cerebrospinal fluid (aCSF), control siRNA or NLRP3 siRNA. (B) Protein levels of NLRP3 in brain of SE rats after 6-week infusion of artificial cerebrospinal fluid (aCSF), control siRNA, or NLRP3 siRNA. (C) Messenger RNA levels of caspase-1 in brain of SE rats after 6-week infusion of aCSF, control siRNA, or caspase-1 siRNA. (D) Protein levels of caspase-1 in brain of SE rats after 6-week infusion of aCSF, control siRNA or caspase-1 siRNA. Data are expressed as a fold change relative to sham group. Columns represent mean ± standard deviation. n = 6 rats per group. **P* < 0.05 versus control siRNA treatment. (E) Protein levels of NLRP3 in brain of sham rats after 6-week infusion of control siRNA, or aCSF. (F) Protein levels of caspase-1 in brain of sham rats after 6-week infusion of control siRNA oraCSF. Data are expressed as a fold change relative to sham group. Columns represent mean ± standard deviation. n = 6 rats per group. NS: not significant versus sham rats. Additional file 3: Table S1. Pump-mediated infusion of small interfering RNA (siRNA) prior to status epilepticus (SE) induction has no potential effects directly on the development and severity of spontaneous recurrent seizures (SRS).

## Abbreviations

SE: status epilepticus
CNS: central nervous system
NLRP3: nucleotide binding and oligomerization domain-like receptor family pyrin domain-containing 3
ASC: apoptosis-associated speck-like protein containing a caspase recruitment domain
siRNA: small interfering RNA
SDS: sodium dodecyl sulfate
PVDF: polyvinylidene fluoride
TRITC: tetramethylrhodamine
SRS: spontaneous recurrent seizures
PCR: polymerase chain reaction
ELISA: enzyme linked immunosorbent assay
TUNEL: terminal deoxynucleotidyl transferase-mediated dUTP end-labeling
RIPA: radio immunoprecipitation assay
EEG: Electroencephalogram
Iba1: ionized calcium binding adaptor molecule 1
BBB: blood–brain barrier
