# Supplementary figures and images for: Inhibition of the NLRP3 inflammasome provides neuroprotection in rats following amygdala kindling-induced status epilepticus
Source: J Neuroinflammation. 2014 Dec 17;11:212. doi: 10.1186/s12974-014-0212-5 (PMC4275944; doi:10.1186/s12974-014-0212-5)

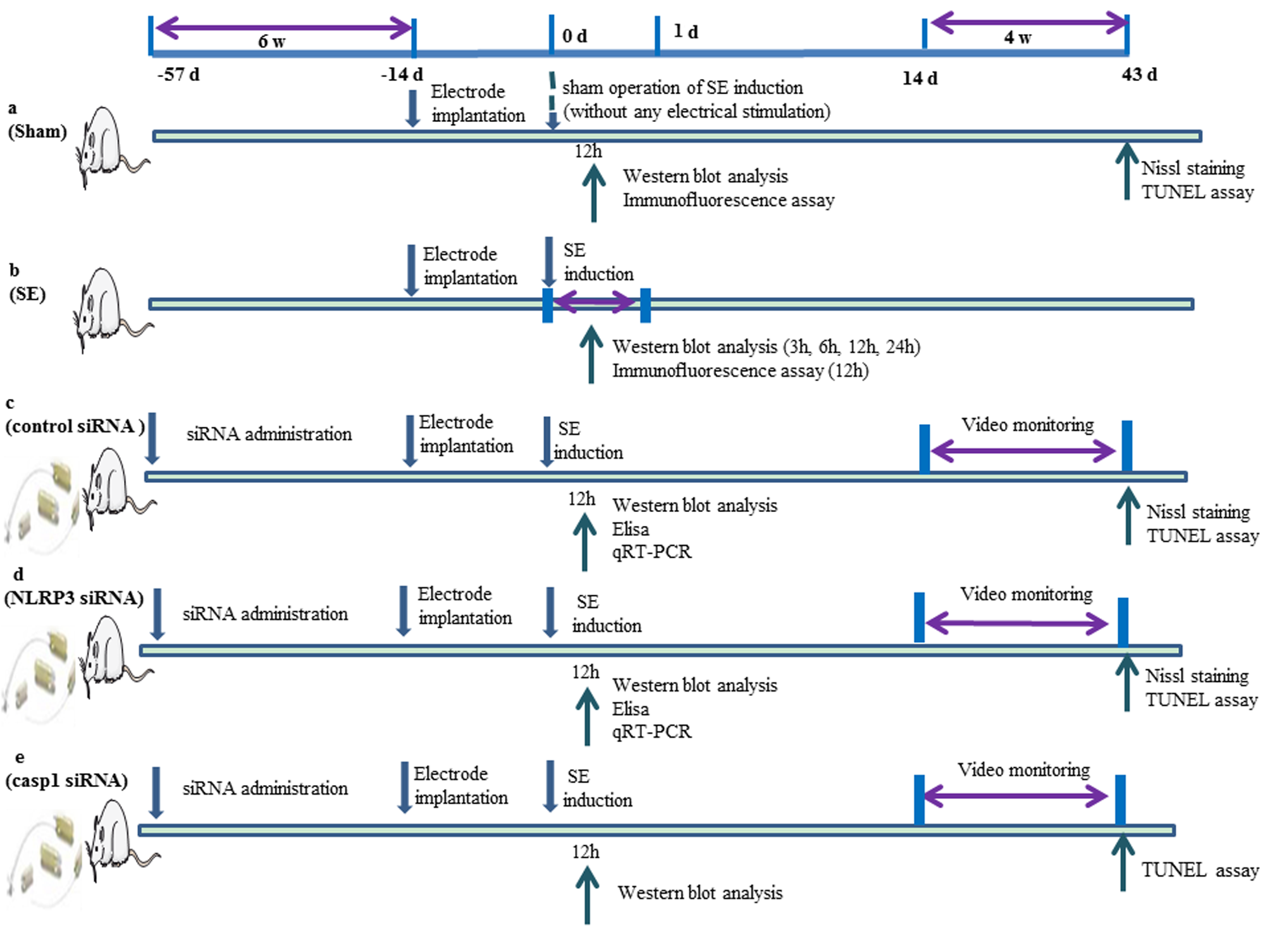

Supplement: Additional file 1: Figure S1. — Scheme of the experimental design and the main experimental protocol. The rats were randomly divided into five groups: sham group, SE group, control siRNA + SE group, NLRP3 siRNA + SE group, and caspase-1 siRNA + SE group. siRNA, small interfering RNA; SE, status epilepticus; TUNEL assay, terminal deoxynucleotidyl transferase-mediated dUTP end-labeling assay; Elisa, enzyme-linked immunosorbent assay; qRT-PCR, quantitative real-time PCR. [file 12974_2014_212_MOESM1_ESM.tiff]

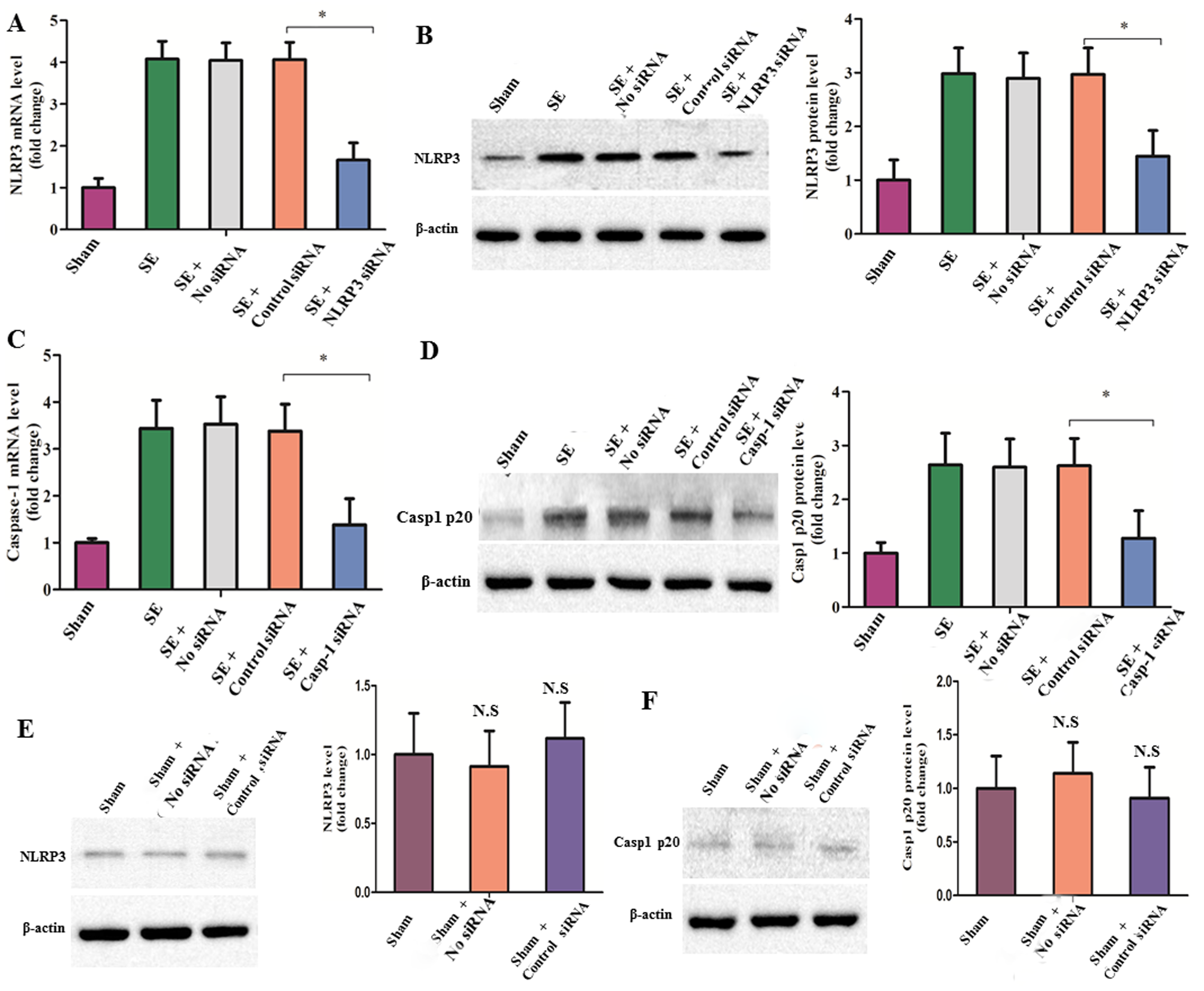

Supplement: Additional file 2: Figure S2. — Small interfering RNA (siRNA) targeting NLRP3 or caspase-1 effectively downregulated NLRP3 or caspase-1 in status epilepticus (SE) rat. (A) Messenger RNA levels of NLRP3 in brain of SE rats after 6-week infusion of artificial cerebrospinal fluid (aCSF), control siRNA or NLRP3 siRNA. (B) Protein levels of NLRP3 in brain of SE rats after 6-week infusion of artificial cerebrospinal fluid (aCSF), control siRNA, or NLRP3 siRNA. (C) Messenger RNA levels of caspase-1 in brain of SE rats after 6-week infusion of aCSF, control siRNA, or caspase-1 siRNA. (D) Protein levels of caspase-1 in brain of SE rats after 6-week infusion of aCSF, control siRNA or caspase-1 siRNA. Data are expressed as a fold change relative to sham group. Columns represent mean ± standard deviation. n = 6 rats per group. *P < 0.05 versus control siRNA treatment. (E) Protein levels of NLRP3 in brain of sham rats after 6-week infusion of control siRNA, or aCSF. (F) Protein levels of caspase-1 in brain of sham rats after 6-week infusion of control siRNA oraCSF. Data are expressed as a fold change relative to sham group. Columns represent mean ± standard deviation. n = 6 rats per group. NS: not significant versus sham rats. [file 12974_2014_212_MOESM2_ESM.tiff]
