# Supplementary material for: Inhibition of the NLRP3 inflammasome provides neuroprotection in rats following amygdala kindling-induced status epilepticus
Source: J Neuroinflammation. 2014 Dec 17;11:212. doi: 10.1186/s12974-014-0212-5 (PMC4275944; doi:10.1186/s12974-014-0212-5)
Supplement: Additional file 3: Table S1. — Pump-mediated infusion of small interfering RNA (siRNA) prior to status epilepticus (SE) induction has no potential effects directly on the development and severity of spontaneous recurrent seizures (SRS). [file 12974_2014_212_MOESM3_ESM.docx]

**Table S1. The performed Small interfering RNA (siRNA) prior to SE induction has no potential effects directly on the development and severity of SRS.**

|  | The number of rats  developed SRS | The time to development  of SRS (days) | The mean number of  seizures (seizures/day) | The mean seizure  duration (sec/seizure) |
| --- | --- | --- | --- | --- |
| SE | 18 (18) | 14.9 ± 5.9 | 7.88 ± 1.12 | 24.91 ± 7.02 |
| No siRNA + SE | 17 (18) ^NS^ | 15.4 ± 5.1 ^NS^ | 8.19 ± 1.06 ^NS^ | 27.57 ± 5.64 ^NS^ |
| Control siRNA + SE | 18 (18) ^NS^ | 16.1 ± 4.5 ^NS^ | 9.01 ± 1.24 ^NS^ | 26.31 ± 5.89 ^NS^ |

All data are shown as mean ± standard deviation (n = 18 per group). ^NS^ P > 0.05, there were not statistically significant differences compared with the SE group.

SRS, spontaneous recurrent seizures; SE, status epilepticus, sec, seconds.
